# Microbiologically Induced Concrete Corrosion: Mechanisms, Key Microorganisms, and Protection Strategies

**DOI:** 10.3390/microorganisms14071425

**Published:** 2026-06-29

**Authors:** Shengxun Yao, Congtao Sun, Yan Wang

**Affiliations:** 1 College of Materials Science and Engineering, Xi’an University of Architecture and Technology, Xi’an 710055, China; yshxhc@163.com; 2 Guangxi Laboratory of Oceanography, Guangxi Academy of Marine Sciences, Guangxi Academy of Sciences, Nanning 530007, China; 3 Key Laboratory of Marine Environmental Corrosion and Bio-Fouling, Institute of Oceanology, Chinese Academy of Sciences, Qingdao 266071, China

**Keywords:** microbiologically induced concrete corrosion, MICC, corrosion mechanism, protection strategy, durability of concrete

## Abstract

Microbiologically induced concrete corrosion (MICC) poses a severe challenge to the long-term durability of infrastructure, particularly in sewer networks and marine environments, which is driven by microbial metabolic activities that attack cement hydrates (Ca(OH)_2_, C-S-H) mainly caused by biogenic sulfuric acid (from sulfur-oxidizing bacteria) or organic acids (from fungi), converting them into expansive gypsum and ettringite, and then cause cracking and spalling. This article reviews advances in mechanisms, key microorganisms, and protection strategies of MICC to enhance our understanding of MICC and provide a guideline for effective protection. The corrosion mechanisms differ by environment: sewers exhibit three-stage pH-driven succession, marine biofilms can either accelerate or inhibit corrosion, while fungi dominate in agricultural and historical settings. Core functional microorganisms involved in MICC include sulfur-oxidizing bacteria (SOB), sulfate-reducing bacteria (SRB), and acid-producing fungi (AF), following pH-dependent succession, while indicator microorganisms for protection efficacy include typical SOB, SRB, and AF that are involved in MICC, as well as general antimicrobial indicator strains (e.g., *Escherichia coli* and *Staphylococcus aureus*) which are used only to assess broad antimicrobial activity and do not represent MICC-specific resistance. Multi-scale deterioration proceeds from microstructural decalcification and pore coarsening to macroscopic mass loss and compressive strength reduction. Protection strategies are categorized into: (i) corrosion-resistant materials (e.g., calcium aluminate cement and alkali-activated materials), (ii) antimicrobial additives (e.g., nano-ZnO and Cu_2_O), (iii) surface coatings (e.g., superhydrophobic coatings and electrodeposited Cu/Cu_2_O layers), and (iv) ecological regulation. However, significant gaps remain between laboratory efficacy and field performance, highlighting the need for long-term validation, multi-scale characterization, intelligent responsive materials, eco-compatible protection systems, and standardized microbial exposure systems.

## 1. Introduction

Concrete is one of the most widely used materials in modern infrastructure construction, and its long-term durability is crucial for its service safety and operational lifespan. However, in specific service environments, concrete structures can be eroded by microorganisms, leading to the deterioration of material properties, a reduction in service life, and even structural safety issues. This phenomenon is known as microbiologically induced concrete corrosion (MICC) [1,2]. MICC represents a cutting-edge, multidisciplinary research topic, involving fields such as microbiology, materials science, environmental engineering, and structural engineering [3]. Sewer networks, due to the abundance of nutrients and sulfur compounds, represent one of the most typical and severe service environments for MICC [1]. Beyond sewer systems, concrete in service environments such as marine settings, agricultural facilities, roadways, historical buildings, and even nuclear waste repositories also faces the threat of deterioration from MICC [4,5].

The essence of MICC is a progressive deterioration process whereby specific microbial communities, through their metabolic activities, progressively modify the micro-environment at the concrete surface, ultimately driving material degradation that spans from microstructural decomposition to macroscopic performance failure [6]. The key functional microorganisms primarily include sulfur-oxidizing bacteria (SOB), sulfate-reducing bacteria (SRB), and acid-producing fungi (AF). Among these, SOB mainly produce sulfuric acid, whereas fungi primarily secrete organic acids (e.g., citric, oxalic), and SRB generate sulfide/H_2_S, which serves as the electron donor for SOB. This acidification reduces the interfacial pH, which in turn dissolves cement hydration products like portlandite (CH) and calcium silicate hydrate (C-S-H) gel and generates expansive products such as gypsum and ettringite (AFt). The crystallization pressure from these products internally leads to concrete cracking and spalling [7,8]. A critical limitation of current mechanistic understanding is that most evidence is derived from pure-culture studies using model organisms such as *Acidithiobacillus thiooxidans*, which may not accurately represent the synergistic and competitive interactions occurring in natural biofilms. This gap has significant practical implications; for instance, it has been reported that cement mortar specimens exposed to single strains of *Acidithiobacillus thiooxidans* or *Halothiobacillus neapolitanus* exhibited approximately a 28% reduction in compressive strength at 300 days, whereas exposure to a mixed consortium resulted in a 52% reduction [9]. This difference undoubtedly underscores the importance of community-level interactions in accelerating corrosion. Such synergistic and competitive interactions among community members have also been confirmed in field and laboratory studies: Okabe et al. [10] demonstrated that SRB activity peaks in a narrow anaerobic zone 150–300 μm below the biofilm surface, directly coupled with an overlying sulfide oxidation zone; Satoh et al. [11] further confirmed this SRB–SOB coupling in a sewer system. Beyond these well-established sulfur-cycling consortia, extracellular polymeric substances (EPS) create physico-chemical gradients that modulate local corrosion microenvironments [12], and fungal–bacterial co-colonization exhibits metabolic interdependencies that are inevitably lost in pure cultures [13]. These findings highlight that a community-level perspective is essential for understanding MICC mechanisms and for developing realistic prediction and mitigation strategies.

The complexity of MICC lies in the fact that the structure, function, and succession patterns of microbial communities vary across different environments, thereby driving distinct MICC mechanisms in different service conditions. However, as we argue throughout this review, the field lacks systematic comparative studies that enable direct cross-environmental predictions. Most studies are conducted in isolation, using different experimental conditions, exposure times, and assessment metrics, making it difficult to synthesize findings into a coherent framework. For instance, in sewage environments, SRB and SOB are spatially coupled, SRB thrive in anaerobic zones (sediment or inner biofilm layers), while SOB dominate oxygenated surface films; this spatial separation is maintained by oxygen and sulfide concentration gradients, continuously producing biogenic sulfuric acid that reacts with cement hydration products to form expansive compounds, causing concrete cracking, spalling, and strength deterioration [1]. In marine environments, complex biofilms composed of bacteria and algae act synergistically with chemical attack to accelerate concrete deterioration [14,15]. In service environments like the atmosphere, agricultural settings, or historical buildings, organic acids secreted by fungi play a major corrosive role [16,17]. Moreover, contrary to the corrosive role of microorganisms, certain biofilms can inhibit concrete corrosion. For example, some biofilms in sewage have been shown to exhibit inhibitory effects on corrosion, as the biofilm can act as a barrier against sewage corrosion to varying degrees [18]. In seawater, it has been found that *Pseudoalteromonas* (B18) and *Paracoccus marcusii* (B23) significantly slow the diffusion of chloride ions (Cl^−^) and magnesium ions (Mg^2+^) into mortar, as well as the leaching of hydroxide ions (OH^−^) from it, thereby increasing the durability of concrete structures in marine environments [19]. Additionally, microbial mineralization has also been reported to inhibit microbial corrosion OF CONCRETE, but this method needs the supply of urea, calcium sources, and other amendments, and its long-term durability remains to be further investigated and validated [20]. Understanding these environment-specific microbial succession patterns and the corresponding corrosion mechanisms is an indispensable theoretical foundation for developing highly efficient, environmentally targeted protective materials and technologies.

Facing the severe challenge of MICC, traditional protection strategies based solely on enhancing material density or simple acid resistance have proven insufficient. Critically, the evidence base for many emerging protection strategies is limited to short-term laboratory tests (typically weeks to months) under idealized conditions, with very few studies extending beyond one year or incorporating real-world environmental fluctuations. This represents a major risk for engineering applications. In recent years, research focus, particularly in understanding MICC mechanisms and materials design, has been shifting towards active and intelligent protection systems [1,21]. However, we identify a significant disconnect between academic research priorities and practical engineering needs: while intelligent and responsive materials receive substantial research attention, their technical complexity and cost currently preclude widespread adoption. Meanwhile, simpler, more deployable strategies remain understudied.

Despite decades of research and hundreds of published studies, the field of MICC protection suffers from several critical limitations, including a persistent gap between laboratory findings and field performance, a lack of standardized testing protocols that hinders cross-study comparison, insufficient attention to long-term durability and life-cycle costs of protection strategies, and an over-reliance on simplified mono-culture systems that poorly represent complex natural microbial communities. These limitations have significantly impeded the translation of fundamental knowledge into effective engineering solutions.

To address these challenges and provide a structured overview, this review critically synthesizes advances in the mechanisms, key microorganisms, and protection strategies of MICC. The review was compiled through a systematic literature search conducted in the Web of Science Core Collection, Scopus, and Google Scholar databases, covering publications from 1990 to 2024. The search strategy employed combinations of keywords related to microorganisms (e.g., “microbial,” “bacteria,” “fungi,” “algae,” “archaea”), material types (e.g., “concrete,” “cement,” “mortar”), and deterioration processes (e.g., “corrosion,” “deterioration,” “biodeterioration,” “microbiologically induced concrete corrosion”). Only peer-reviewed journal articles, conference proceedings, and authoritative technical reports were considered. Additional relevant studies were identified by cross-referencing the bibliographies of selected papers. Articles were screened by title and abstract, and full-text review was conducted for studies meeting the preliminary inclusion criteria; studies reporting purely chemical corrosion without biological involvement were excluded.

Specifically, this review aims to: (i) analyzes the chemo-biological mechanisms and corrosion characteristics of MICC across different service environments; (ii) elucidate the core microbial communities that drive MICC, as well as the indicator microorganisms used for evaluating protection efficacy; (iii) clarify the multi-scale deterioration pathways from microstructural failure to macroscopic performance degradation caused by microorganism-induced concrete damage; and (iv) review recent progress in protection strategies, categorized into corrosion-resistant matrix materials, antimicrobial additives, surface coatings, and ecological regulation. Finally, by integrating multidisciplinary perspectives and extensive literature evidence, we identify key scientific questions and technical challenges, offering constructive recommendations for future research and engineering practice.

## 2. Mechanisms of MICC and Key Microorganisms

### 2.1. Mechanisms of MICC in Sewage Environments

**Figure 1:**
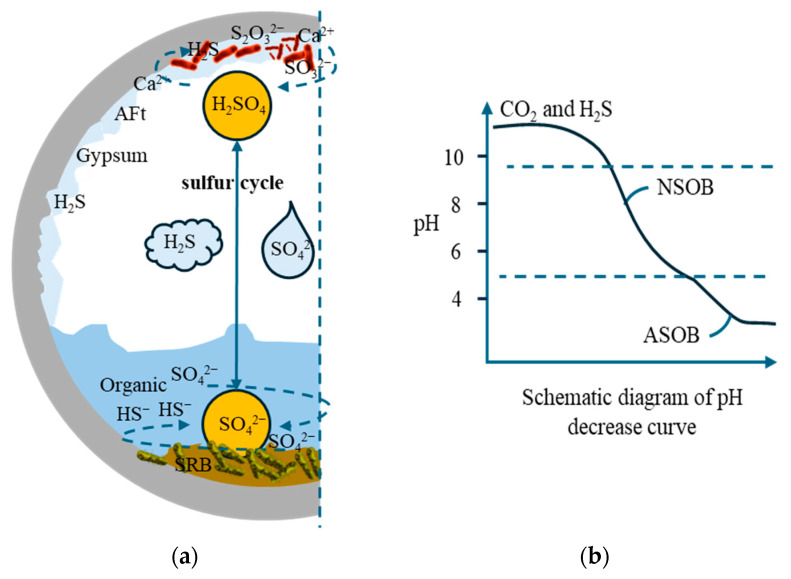
Diagram of the MICC mechanism in sewer environments, (**a**) main events involved in MICC (modified from [2]), (**b**) main three stages of MICC (modified from [7,19]). Dotted lines with arrows: micro-zone biogeochemical cycles.

In sewer networks, MICC is primarily driven by the sulfur cycle. It is a complex, multi-stage process involving the synergistic action of various microbial groups, accompanied by the succession of microbial community composition (Figure 1). For spatial distribution (Figure 1a), distinct niches are formed in different parts of the pipeline (crown, wall, invert) due to variations in humidity, H_2_S concentration, and nutrient composition. The investigation of 16S rRNA gene amplicon sequencing showed [22,23] that the gaseous phase at the crown and upper walls is primarily colonized by SOB, such as *Acidithiobacillus*, *Halothiobacillus*, and *Thiomonas*, while the sediment and liquid phase at the invert are dominated by SRB like *Desulfomicrobium*, *Desulfobulbus,* and fermentative bacteria like *Macellibacteroides*. These were consistent with the results of cultivation [24] and the oligonucleotide probes [25] method.

As for temporal succession (Figure 1b), the corrosion process strictly follows a pH gradient, exhibiting a typical three-stage process: (1) surface carbonation and neutralization (surface pH between 10 and 11), (2) establishment of a microbial biofilm (surface pH between 4 and 9), and (3) severe corrosion where surface pH can drop below 1 [7,26]. Initially, the environment, rich in organic matter and sulfates, provides ideal anaerobic conditions for SRB metabolism. These bacteria proliferate in the sediments at the pipe invert or within the inner layers of biofilms, producing copious amounts of H_2_S. This gaseous H_2_S then diffuses into the upper gas phase of the pipe and dissolves into the condensate film on the pipe wall, coupled with carbonation, the pH of the concrete begins to decrease from its initial alkaline state [1,7,26].

The main biochemical processes are as (1)–(4) chemical equations, where CH_2_O represents organic matter.

(1) $${{SO}_{4}}^{2-}+2CH_{2}O\overset{SRB}{\to }H_{2}S+2{{HCO}_{3}}^{-}$$

(2) $$H_{2}S\to S^{2-}+2H^{+}$$

(3) $$CO_{2}+H_{2}O\to {{CO}_{3}}^{2-}+2H^{+}$$

(4) $$2H^{+}+Ca(OH{)}_{2}\to {Ca}^{2+}+2H_{2}O$$

When the surface pH of the concrete drops to approximately 9, neutrophilic sulfur-oxidizing bacteria (NSOB), such as *Halothiobacillus neapolitanus*, begin to attach and form a biofilm [1,7,26]. These bacteria oxidize reduced sulfur compounds (H_2_S, S_0_, and S_2_O_3_^2−^) into biogenic sulfuric acid, making MICC the dominant process governing concrete deterioration. Consequently, the concrete surface pH continues to decrease from its alkaline state, and the acid attack intensifies [27,28]. As the pH falls below 4, acidophilic sulfur-oxidizing bacteria (ASOB), such as *Acidithiobacillus thiooxidans* and *A. ferrooxidans*, become the dominant microbial consortium. These microorganisms can produce extremely high concentrations of sulfuric acid, with the capacity to lower the pH to below 1, thereby triggering severe chemical corrosion [1,7,24,26]. This progressive acidification is initially driven by neutrophilic SOB, which produce acid under near-neutral conditions and gradually lower the pH to levels that favor acidophilic SOB; the latter then dominate due to their higher acid tolerance and metabolic efficiency at pH < 4 [7,24,26]. The biogenic sulfuric acid thus generated chemically reacts with the cement hydration products. This includes neutralizing Ca(OH)_2_ in the pore solution to form gypsum (CaSO_4_·2H_2_O). In addition, sulfuric acid reacts with calcium aluminate phases (e.g., tricalcium aluminate, C_3_A) present in the cement, leading to the formation of ettringite (AFt). However, ettringite formation is not universal; it depends critically on the availability of reactive aluminate phases in the binder composition, as well as environmental conditions such as sulfate concentration, pH, and moisture availability. The expansive stresses generated by the crystallization of gypsum and ettringite lead to concrete cracking, which in turn accelerates the penetration of aggressive agents and exacerbates structural deterioration [29,30,31].

The main biochemical processes are represented by chemical Equations (5)–(10).

(5) $$H_{2}S+2O_{2}{\;\overset{SOB}{\to }H}_{2}SO_{4}$$

(6) $$H_{2}SO_{4}\to 2H^{+}+{{SO}_{4}}^{2-}$$

(7) $$2H^{+}+Ca{\left(OH\right)}_{2}\to Ca^{2+}+2H_{2}O$$

(8) $${{SO}_{4}}^{2-}+Ca^{2+}+2H_{2}O\to CaSO_{4}\cdot 2H_{2}O$$

(9) $$H_{2}SO_{4}+{CaCO}_{3}\to CaSO_{4}+H_{2}CO_{3}$$

(10) $$3CaO\cdot Al_{2}O_{3}+3CaSO_{4}\cdot 2H_{2}O+26H_{2}O\to 3CaO\cdot Al_{2}O_{3}\cdot 3CaSO_{4}\cdot 32H_{2}CO_{3}$$

**Figure 2:**
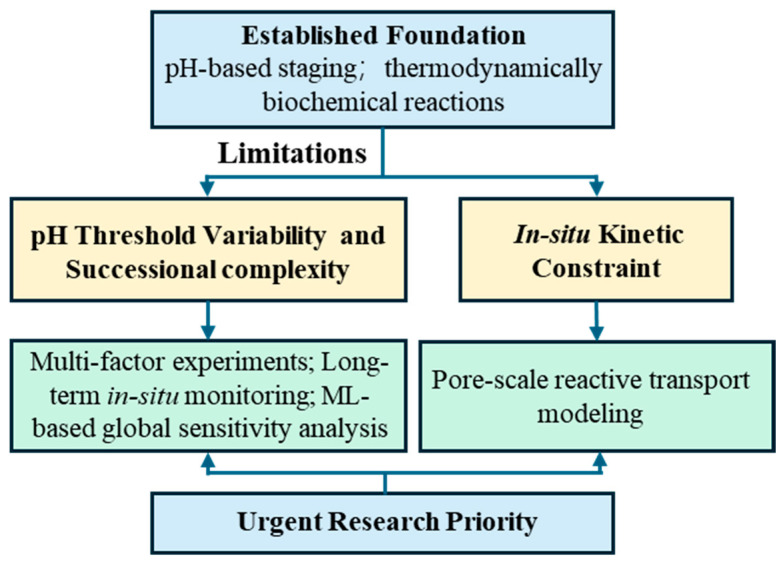
Conceptual framework of knowledge gaps and future research priorities for MICC in sewage environments.

Therefore, MICC in sewage environments represents a typical example of ecosystem engineering, where the destructive power arises from the spatial and temporal succession and synergistic interactions among diverse functional microorganisms, coupled with their metabolic activities and the associated elemental biogeochemical cycling. While this pH-based staging is widely accepted, the precise pH thresholds at which community shifts occur vary across studies, likely due to differences in microbial community composition, nutrient availability, and concrete surface properties. Moreover, the assumption of unidirectional succession may be oversimplified, given the complexity of the actual service environment. Future work should employ machine learning (ML)-based global sensitivity analysis to decouple the confounding effects of multiple environmental covariates on the observed pH-threshold variability. Moreover, the biochemical reactions presented (Equations (1)–(8)) are thermodynamically well-established. However, the in situ rates of these reactions within concrete pore structures remain poorly constrained. Most rate data come from batch culture studies or idealized flow cells, which may not accurately reflect the diffusion-limited conditions within partially degraded concrete. Figure 2 presents a conceptual framework of knowledge gaps and future research priorities for MICC in sewage environments.

### 2.2. Mechanisms of MICC in Marine Environments

The corrosion of concrete in marine environments results from the combined action of chemical attack and microbial activity, rendering its mechanism more complex. The high concentrations of chloride ions, sulfate ions, and magnesium ions in seawater themselves constitute chemical corrosion. Additionally, the microorganisms, primarily bacteria, algae, and fungi, attach to the concrete surface, forming biofilms that significantly alter the local micro-environment and accelerate deterioration [19,32,33]. The microorganisms involved in MICC within marine biofilms primarily include SOB, SRB, and photosynthetic microorganisms like cyanobacteria, green algae, and diatoms [14,32,34]. In aerobic zones, SOB, such as *Thiobacillus* spp., oxidize reduced sulfur compounds like H_2_S, S_0_, S_2_O_3_^2−^, etc., present in seawater to sulfuric acid, leading to a pH decrease on the concrete surface and the formation of gypsum [15,31]. It should be noted that the *Thiobacillus* genus used here did not directly originate from the concrete surface in the marine environment, though numerous studies have confirmed the existence of marine sulfur-oxidizing groups such as *Thiobacillus thioparus* in the marine environment sediment; there is currently no direct evidence of their involvement in the MICC in natural sea areas [35,36]. Algae, such as Enteromorpha and diatoms, proliferate extensively in the tidal and splash zones. Their photosynthetic activity alters the surface pH, and the organic acids secreted during metabolism can dissolve the calcareous components of the cement matrix, thereby modifying the microstructure of the concrete [14]. A critical unresolved question is whether microbial activity merely accelerates purely chemical corrosion or whether qualitatively distinct deterioration mechanisms operate in the presence of biofilms, and how the contribution of microorganisms to the deterioration of concrete can be quantified. Moreover, the literature contains contradictory reports on whether marine biofilms are net detrimental or beneficial for concrete durability. Some studies report accelerated corrosion and increased penetration depths, while others observe that dense biofilms impede chloride ingress and reduce reinforcement corrosion [19,33,37,38]. To clarify this apparent contradiction, Table 1 summarizes the contrasting effects of corrosive versus protective biofilms in marine concrete based on biofilm composition, thickness, maturity, and environmental conditions. However, the specific conditions that tip the balance from protection to corrosion have not been systematically investigated. This represents a critical knowledge gap for engineering applications, where the goal may be to promote protective biofilms rather than eliminate all microbial activity.

Furthermore, the progression of MICC in marine environments is regulated by various physicochemical environmental factors. Temperature is a critical driver, as seasonal and spatial thermal variations significantly influence microbial metabolic rates, community composition, and biofilm stability; warmer conditions generally accelerate sulfur oxidation kinetics and SOB activity, thereby promoting microbial corrosion of concrete [38]. High salinity conditions (>3 g/L) promote the metabolism of SRB and accelerate sulfate reduction rates, thus accelerating the sulfur cycle and the generation of gypsum [34,38]. The concentration of dissolved oxygen in seawater is another key factor governing community succession; oxygen-rich zones favor the proliferation of SOB like *Thiobacillus* and ammonia- and nitrite-oxidizing microorganisms like *Nitrosopumilus*, which accelerates biogenic sulfuric acid corrosion and consequently impacts the durability of concrete structures [33,37,39]. Meanwhile, MICC is most pronounced in tidal zones, where wet-dry cycles stimulate biofilm growth and metabolic activity, thereby accelerating corrosion [32]. Nutrient limitation also exerts a strong influence on microbial succession: when organic carbon or essential nutrients (e.g., nitrogen, phosphorus) become scarce, competitive exclusion tends to favor oligotrophic SOB over heterotrophic competitors, thereby altering the functional composition of the biofilm community and potentially modulating corrosion rates, although direct evidence linking these patterns to specific genera or species remains limited.

### 2.3. Mechanisms of MICC in Other Service Environments

Beyond sewer networks and marine engineering, MICC is also observed in a wide range of service environments, including agriculture, nuclear waste disposal, historical buildings, and various industrial facilities. Although the specific mechanisms vary with the environment, they can be attributed to the erosion of concrete by acids produced through microbial metabolism.

In agricultural and industry environments, the continuous input of organic matter fuels corrosion processes linked to microbial metabolism [40]. In livestock manure, silage effluent, or anaerobic digestate, the synergistic action of SRB, acid-producing fermentative bacteria, and SOB generates organic acids (e.g., acetic acid, propionic acid), biogenic sulfuric acid, and CO_2_. This leads to the decomposition of C-S-H gel in the cement matrix and the leaching of calcium ions, resulting in strength loss and a loosened structure [40,41,42]. In anaerobic digestion facilities, microbial succession is driven by the fermentation substrate, with initial dominance by acid-producing bacteria like *Clostridium*, later succeeded by a mixed consortium of SOB like *Halothiobacillus neapolitanus*, methanogenic archaea, and SRB [41,42,43].

On the surfaces of historical buildings, influenced by light and humidity, the colonizing community is often dominated by cyanobacteria like *Oscillatoria*, *Lyngbya*, and *Leptolyngbya*; lichenized fungi like *Toninia nordlandica*, *Lobaria quercizans*, *Lecanora subcarnea*, and *Cystocoleus ebeneus*; and fungi from the Ascomycota phylum such as *Aspergillus niger*, *A. fumigatus*, and the genus of *Penicillium* and *Fusarium* [44,45]. Fungi like *Aspergillus tamarii*, *A. niger*, and *Penicillium commune* often involve both biochemical acidolysis (organic acid secretion) and physical penetration by hyphae, which create microcracks and lead to the deterioration of concrete [46,47]. Damp-surface algae are also implicated in biodeterioration, resulting in aesthetic, physical, and chemical damage to the facades of the surveyed buildings [44].

In nuclear repositories and other buildings. Although the highly alkaline conditions (pH > 12) inhibit most microorganisms, indigenous alkaliphilic or alkali-tolerant bacteria, such as SRB and methanogens, present in the clay and groundwater can still metabolize organic matter to produce H_2_S and organic acids, potentially threatening the long-term stability of concrete barriers [5,48]. In freshwater hydraulic structures, algae, actinomycetes, and sulfur-metabolizing bacteria form biofilms on the concrete surface. Through photosynthetic alteration of local pH or sulfur-cycle-mediated sulfuric acid production, they cause surface corrosion and strength loss [49,50]. In other buildings, such as bridges, cooling towers, and geothermal power plants, SOB like *Acidithiobacillus ferrooxidans* can also oxidize reduced sulfur compounds to sulfuric acid, leading to MICC [51,52].

**Figure 3:**
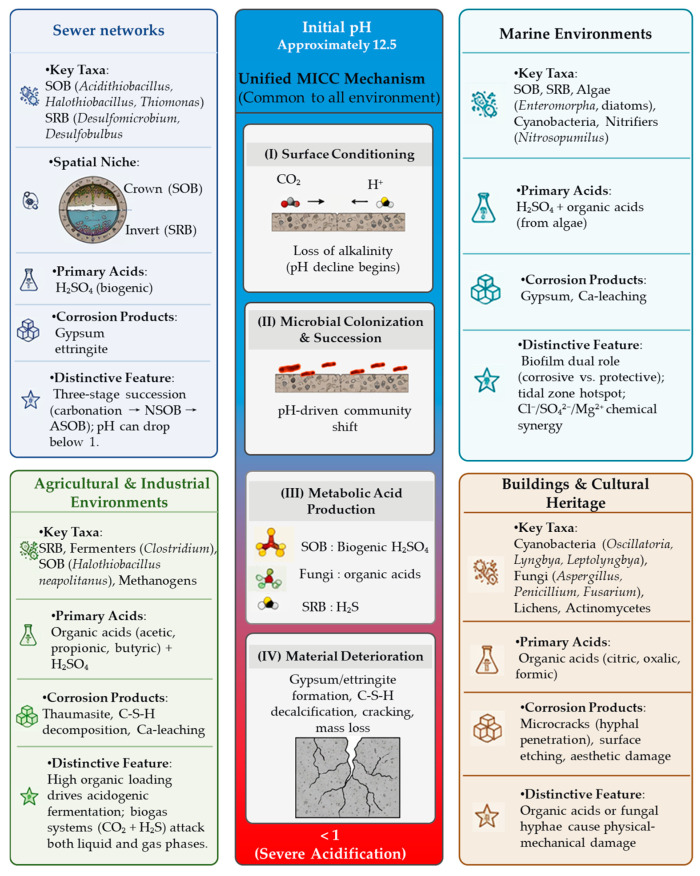
Diagram of microbially induced concrete corrosion (MICC) mechanisms across diverse service environments.

Overall, despite the diverse environmental contexts, these corrosion processes generally follow a common pathway: microbial colonization, metabolic acid production, dissolution of hydration products, formation of expansive products, and structural deterioration (Figure 3).

### 2.4. Key Microorganisms in MICC Mechanism Research

To elucidate the mechanisms of MICC, researchers have conducted extensive studies ranging from field investigations to laboratory simulations, involving a complex array of microbial taxa. Table 2 lists primary microbial groups implicated in MICC as reported.

Microorganisms Involved in the Sulfur Biogeochemical Cycle. Among these, SOB represents the most intensively studied model microorganisms. Common genera include *Acidithiobacillus*, such as *A. thiooxidans* [31,42,53,54,55,56] and *A. ferrooxidans* [51,57,58,59]. These are widely employed to investigate the mechanisms of sulfuric acid generation under low pH conditions and its subsequent aggressive action on various cementitious materials like ordinary Portland cement and alkali-activated materials [60]. *Halothiobacillus* species, notably *H. neapolitanus* [61,62,63], along with *Thiobacillus* species such as *T. thioparus* [15,31] and *T. intermedius* [28,42,53], are primarily used in studies focusing on MICC mechanisms during neutral to acidic transitional phases. Furthermore, *Starkeya novella* [28] has been utilized in research concerning bacterial calcium ion transport during MICC, while *Streptomyces* [52], possessing sulfur oxidation capabilities, has garnered attention in some investigations. Additionally, SRB play a crucial role by reducing sulfate to H_2_S in anaerobic niches, thereby supplying reduced sulfur sources for SOB and establishing a synergistic corrosion mechanism. Representative genera include *Desulfovibrio*, used to study metabolic activity in simulated concrete pore solutions and its corrosive effects on steel reinforcement [64]; and *Desulfomicrobium* [61] and *Desulfobulbus* [22], which have been detected in sewer biofilms and are implicated in the sulfur cycle.

Beyond taxonomic characterization, functional genes have emerged as powerful molecular markers for profiling sulfur-cycle microorganisms in environmental samples. Key examples include *dsrAB* (encoding dissimilatory sulfite reductase) for sulfate-reducing prokaryotes, *soxB* (encoding sulfate thioesterase) for sulfur-oxidizing bacteria, and *aprA* (encoding adenosine-5’-phosphosulfate reductase), which is conserved among both sulfate-reducing and sulfur-oxidizing prokaryotes [65]. The *aprA* gene-based approach has been successfully applied to investigate the diversity of sulfur-cycle prokaryotes in diverse habitats, including sediments and water columns [66,67]. These functional gene markers enable a more mechanistic linkage between microbial community composition and actual sulfur metabolic activities in MICC environments, complementing the taxonomic identification of key genera.

Fungi contribute to concrete corrosion through the secretion of organic acids like citric acid and oxalic acid and through mechanical penetration by hyphae. The organic acids produced are strongly modulated by environmental conditions, particularly the availability and type of carbon sources, relative humidity, ambient pH, and oxygen, which are critical influencing factors in MICC by fungi [68]. These species commonly belong to the genera such as *Aspergillus*, including *A. niger* [16,22,46,69], *A. fumigatus* [44], *A. tamarii* [47], and *A. versicolor* [70]; *Penicillium*, including *P. chrysogenum* [70], *P. commune* [46]; and *Cladosporium*, including *C. herbarum* [70], *C. sphaerospermum* [46,68,69]. These fungi are frequently isolated from deteriorated concrete surfaces [46,69] and are commonly employed to investigate mechanisms of biofilm formation and their role in concrete surface degradation [16,47,68]. Other fungal genera, such as *Paecilomyces*, *Stemphylium* [16], and *Trichoderma* [69], have also been reported in some studies.

In environments with sufficient light, photosynthetic microorganisms form biofilms that influence concrete surface properties. Representative species include cyanobacteria, such as *Oscillatoria* and *Lyngbya*, which have been detected on various concrete surfaces and are involved in biofilm formation and material deterioration [45,50]. Additionally, macroalgae like Enteromorpha and diatoms [14] have also been researched to assess their impact on mortar properties in marine environments.

Investigating these key microbial participants in MICC allows researchers to move beyond the complexity of natural communities and focus on the specific mechanisms of interaction between individual microorganisms and materials. This approach provides a fundamental basis for developing and validating theoretical models, such as reactive-transport models. However, most mechanistic studies have employed pure cultures of a small number of model organisms, primarily *A thiooxidans*, *A. ferrooxidans*, and *A. niger*. This approach has yielded valuable mechanistic insights; it has significant limitations, for it cannot capture synergistic or competitive interactions among community members; it may overestimate or underestimate corrosion rates depending on whether key community interactions are missing; and the metabolic characteristics of laboratory cultures may vary after prolonged laboratory passage. These shortcomings underscore the urgent need for standardized synthetic microbial consortia that better reflect the functional diversity of natural MICC communities, as well as the adoption of biofilm-based test systems that more realistically reproduce the spatial organization, mass-transport constraints, and surface colonization prevalent in actual concrete environments. By combining these two aspects, researchers are expected to be able to systematically disentangle the contributions of individual populations within a community context, while generating robust, mechanistic data for model calibration, ultimately bridging the gap between laboratory simplifications and field complexity.

**Table 2:** Microorganisms reported in mechanistic studies of MICC.

Microbial Category	Genus/Species	References
SOB	*Acidithiobacillus thiooxidans*	[31,42,53,54,55,56]
	*A*. *ferrooxidans*	[51,57,58,59]
	*Halothiobacillus neapolitanus*	[61,62,63]
	*Thiobacillus thioparus*	[15,31]
	*Thiomonas intermedia* (Former name *Thiobacillus intermedius*)	[28,42,53]
	*Starkeya novella*	[28]
	*Streptomyces* spp.	[52]
SRB	*Desulfovibrio desulfuricans*	[64]
	*Desulfomicrobium* spp.	[61]
	*Desulfobulbus* spp.	[22]
Fungi	*Aspergillus niger*	[16,46,69]
	*A*. *tamarii*	[47]
	*A*. *versicolor*	[70]
	*Penicillium chrysogenum*	[70]
	*P*. *commune*	[46]
	*Cladosporium herbarum*	[46,69]
	*C. sphaerospermum*	[46,69]
	*Paecilomyces* spp.	[16]
	*Stemphylium* spp.	[16]
	*Trichoderma atroviride*/*T. harzianum*	[69]
Photosynthetic Microorganisms	*Enteromorpha* spp.	[14]
	diatoms (*Fragilaria*, *Navicula*, *Achnanthes*, etc.)	[14]
	cyanobacteria (*Oscillatoria*, *Lyngbya*, *Leptolyngbya*, etc.)	[45]

### 2.5. Indicator Microorganisms for Evaluating Protection Efficacy

To assess the protective performance of novel antimicrobial concretes, coatings, or admixtures, it is essential to select representative indicator microorganisms that enable reliable evaluation of antimicrobial or anticorrosion efficacy. Based on their functional roles, these test organisms can be divided into two categories: corrosive functional microorganisms, which directly reflect the material’s ability to resist microbiologically induced corrosion (MIC); and general microbial indicators, which are used to evaluate the material’s broad-spectrum antimicrobial properties.

The core indicator microorganisms in the first category are those involved in the key steps of concrete corrosion, namely the sulfur cycle and acid production (Table 3). SOB is the most widely applied functional bacteria.

Acidophilic SOB, such as *Acidithiobacillus thiooxidans* [53,54,56,60,71,72,73,74,75,76,77] and *A. ferrooxidans* [51], are key species for simulating severe corrosive environments. Neutrophilic SOB, including *Halothiobacillus neapolitanus* [62], *Thiomonas intermedia* [53], *Thiobacillus thioparus* [78], and *Starkeya novella* [79], are used to evaluate corrosion potential under initially neutral conditions.

SRB, represented by *Desulfovibrio* spp. [64,73,78,80,81,82] indirectly promote corrosion by producing H_2_S and are frequently employed to evaluate the protective efficacy of materials against MICC under anaerobic or partially anaerobic conditions.

Additionally, acid-producing fungi contribute to concrete corrosion through the secretion of organic acids. Common test species include *Aspergillus niger* [83,84,85], *Penicillium* spp. [86], and *Fusarium oxysporum* [87]. These fungi are routinely employed to evaluate the antifungal resistance of concrete materials, particularly in humid or biofouling-prone environments.

**Table 3:** Corrosive functional microorganisms for evaluating the protective effect against MICC.

Microbial Category	Genus/Species	References
SOB	*Acidithiobacillus thiooxidans*	[53,54,56,60,71,72,73,74,75,76,77]
	*A. ferrooxidans*	[51]
	*Halothiobacillus neapolitanus*	[62]
	*Thiomonas intermedia*	[53]
	*Starkeya novella*	[79]
	*Thiobacillus thioparus*	[78]
SRB	*Desulfovibrio* spp.	[64,73,78,80,82]
Fungi	*Aspergillus. niger*	[83,84,85]
	*Fusarium orysporum*	[87]
	*Penicillium* spp.	[86]

For the latter category, i.e., indicator microorganisms for broad-spectrum antimicrobial properties, model strains that are easy to culture and grow rapidly are typically selected (Table 4). Examples on the bacterial side include the Gram-negative bacterium *Escherichia coli* [78,83,88,89,90,91,92,93,94,95,96] and *Pseudomonas* spp. [94]; and the Gram-positive bacteria *Staphylococcus aureus* [83,90,92,93,94,95] and *Bacillus subtilis* [89]. Regarding fungi, in addition to *Aspergillus niger*, *Candida albicans* [84] and *Saccharomyces cerevisiae* [96] are also used for antimicrobial performance testing. It should be noted that *S. cerevisiae* is included solely as a reference strain for antifungal susceptibility testing; it is not representative of MICC-relevant fungi and therefore cannot inform predictions of microbial corrosion. Likewise, results from standard antibacterial assays using *E. coli*, *S. aureus*, or *C. albicans* do not reliably predict resistance to MICC, for the physiological traits, metabolic pathways, and stress tolerance mechanisms of corrosive functional microorganisms may differ fundamentally from those of these standard laboratory strains.

## 3. Multi-Scale Deterioration Path of Concrete Performance in MICC

### 3.1. Microstructural Damage of Concrete Induced by Microorganisms

**Figure 4:**
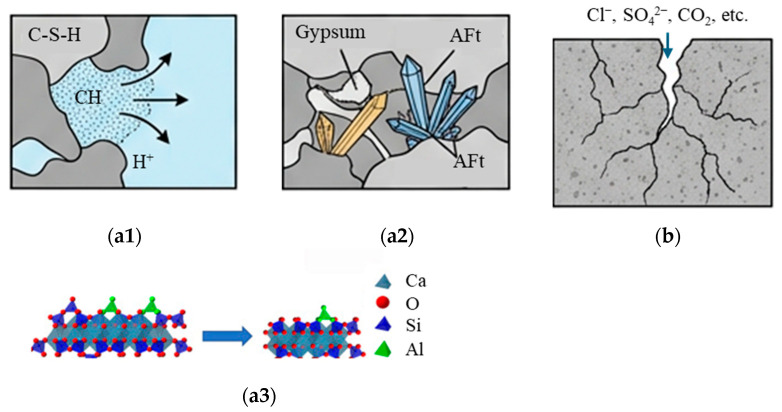
Schematic diagram of microstructure destruction in MICC. (**a1**) Hydration product eroded in concrete; (**a2**) The formation of gypsum and calcium aluminate crystals; (**a3**) The break of C-A-S-H chains (modified from [74]); (**b**) The formation of cracks and erosive ion transport.

At the microscopic scale, the deterioration of concrete caused by MICC is a complex, multi-stage evolutionary process driven by biogeochemical actions [29,51,53]. It is primarily manifested as the decomposition of gel phases, deposition of corrosion products, increased porosity, and surface roughening, which directly impact the material’s durability and service life [6,53,97] (Figure 4).

During the MICC process, microstructural damage manifests, on one hand, as the chemical decomposition of cement hydration products and the leaching of key ions. Biogenic acids neutralize the alkaline substances in the cement paste, leading to the dissolution of portlandite (CH) and calcium silicate hydrate (C-S-H) gel, resulting in substantial leaching of calcium ions (Ca^2+^) [29,51,53]. This decalcification progressively lowers the Ca/Si ratio of the C-S-H phase; as calcium is preferentially removed from the interlayer and bridging positions, the silicate tetrahedral chains undergo depolymerization, ultimately forming a silica-rich amorphous gel with limited binding capacity. The loss of calcium and the corresponding collapse of the C-S-H structure significantly increase matrix porosity and loosen the overall microstructure [15,29,98]. For alkali-activated slag (AAS), following the removal of interlayer CaO polyhedra, the short chains of calcium-aluminosilicate hydrate (C-A-S-H) fracture at the nanoscale, forming a network of silicon-aluminum assemblages. Under microbial corrosion conditions, the Al tetrahedra at the bridging sites of C-A-S-H in AAS pastes are more readily removed than Si tetrahedra [74]. In alkali-activated fly ash (AAF) mortars, corrosion leads to the leaching of aluminum and sodium ions, increasing porosity. Conversely, in alkali-activated slag (AAS) mortars, the excessive formation of gypsum contributes to an increase in large capillary pores [56,99].

On the other hand, the formation and volume expansion of MICC products drive the destruction of the concrete microstructure. Research indicated that dissolved Ca^2+^ reacts with SO_4_^2−^ to form expansive secondary minerals such as gypsum (CaSO_4_·2H_2_O), and SO_4_^2−^ reacts with tricalcium aluminate (C3A) to form ettringite (AFt). The molar volumes of these products are larger than those of the original components, generating crystallization pressure that induces microcracks and macroscopic cracking [29,55,100]. For instance, extensive cracking has been observed in blast furnace slag cement mortar linings due to the accumulation of corrosion products like ettringite [29]. Concurrently, the coarsening of the pore structure and the interconnection of microcracks significantly increase the permeability of aggressive ions (e.g., Cl^−^, SO_4_^2−^) and gases (e.g., CO_2_, O_2_), thereby accelerating concrete corrosion.

Microstructural damage in concrete is the fundamental cause of the deterioration of its macroscopic properties. While the microstructural changes induced by MICC have been extensively characterized using techniques such as SEM, XRD, and mercury intrusion porosimetry, the continuous linkage between nanoscale C-S-H depolymerization, microscale pore coarsening, and macroscopic mechanical degradation remains poorly understood. Most studies report endpoint characterizations at specific time points, but the dynamic evolution of microstructural damage, particularly the critical thresholds at which damage becomes irreversible or accelerates, has not been systematically quantified. This limits the development of predictive service life models. To address this gap, dynamic monitoring techniques should be employed, including micro-CT for three-dimensional pore structure evolution, Raman mapping for chemical phase distribution, SEM-EDS time-series analysis for tracking elemental changes at identical locations over time, and in situ pH profiling for real-time surface acidity monitoring at the biofilm–concrete interface [101,102]. Moreover, the decalcification of C-S-H and the formation of gypsum and ettringite are well-established as the primary microstructural degradation mechanisms. However, the relative importance of these mechanisms may vary with concrete composition and exposure conditions.

### 3.2. Macroscopic Performance Deterioration of Concrete Induced by MICC

The cumulative effect of microstructural damage ultimately manifests as the deterioration of macroscopic properties. MICC-induced macroscopic performance deterioration is characterized by significant degradation in the physical form and mechanical properties of the concrete, which can be comprehensively characterized by macroscopic indicators such as mass loss, strength reduction, surface morphology degradation, and a decrease in the overall load-bearing capacity of the structure [103,104,105]. Critically, these material-level responses are preceded and driven by measurable microbiological and chemical shifts—specifically, a progressive drop in surface pH and the accumulation of sulfate within the pore solution, which promote the precipitation of gypsum and secondary ettringite. Thus, pH decline and sulfate build-up usually serve as early-stage proxies that mechanistically link microbial sulfur oxidation to the ensuing macroscopic deterioration [29,55,100].

Surface morphology degradation is the most significant macroscopic feature of MICC. MICC frequently leads to surface cracking, powdering, spalling, and the formation of etch pits, accompanied by softening, sanding, algal deposits, and the accumulation of white or yellowish-brown pasty products (primarily gypsum) [58,59,106]. Macroscopically, the consequences of MICC often exhibit a layered development, which can be divided from the exterior to the interior into a severely deteriorated layer, a separation layer, and visible and invisible transition layers. The corrosion depth intuitively reflects the extent of aggressive media ingress. For instance, after 8 months of corrosion in seawater containing SOB, the neutralization depth of specimens in the biotic test group reached 12.4 mm, approximately 2.3 times that of the control group [15]. Some studies have found that the corrosion front profile and the corrosion depth follow a square root trend over time [107].

Mass loss is one of the most direct indicators of macroscopic performance deterioration in MICC. MICC causes the dissolution of calcium hydroxide and C-S-H gel in concrete, leading to calcium ion leaching and matrix degradation [6,53,98]. Simultaneously, the crystallization and expansion of corrosion products (such as gypsum and ettringite) induce internal cracking and surface spalling, resulting in direct physical loss of material [29,55,100]. The synergistic action of these biochemical and physical processes ultimately results in a significant reduction in concrete mass and a deterioration of structural integrity. For example, after long-term exposure, the mass loss rate of concrete can reach 2.96~4.65%, with microbial action contributing approximately 32.3~36.6% of the total loss [49].

Mechanical property degradation is another key manifestation of MICC, especially the reduction in compressive strength. MICC increases concrete porosity, leading to a decrease in mechanical properties such as compressive strength and flexural strength [7]. In the initial stages, the compressive strength of mortar may temporarily increase due to the formation of expansive corrosion products like gypsum and calcium carbonate; however, as MICC progresses, the compressive strength subsequently decreases [108]. As discussed in the Introduction, MICC results in compressive strength reduction, and mixed-consortium exposure resulted in significantly greater compressive strength loss than single-strain exposure [9].

Furthermore, the synergistic interaction between steel reinforcement corrosion and concrete MICC deterioration is particularly concerning for structural safety [97], yet it remains severely understudied. Most MICC studies use unreinforced concrete specimens, ignoring the coupled electrochemical–mechanical–biochemical processes that occur in real reinforced concrete structures.

## 4. Protection Strategies for MICC

### 4.1. Corrosion-Resistant Cementitious Materials and Concrete Modification

**Figure 5:**
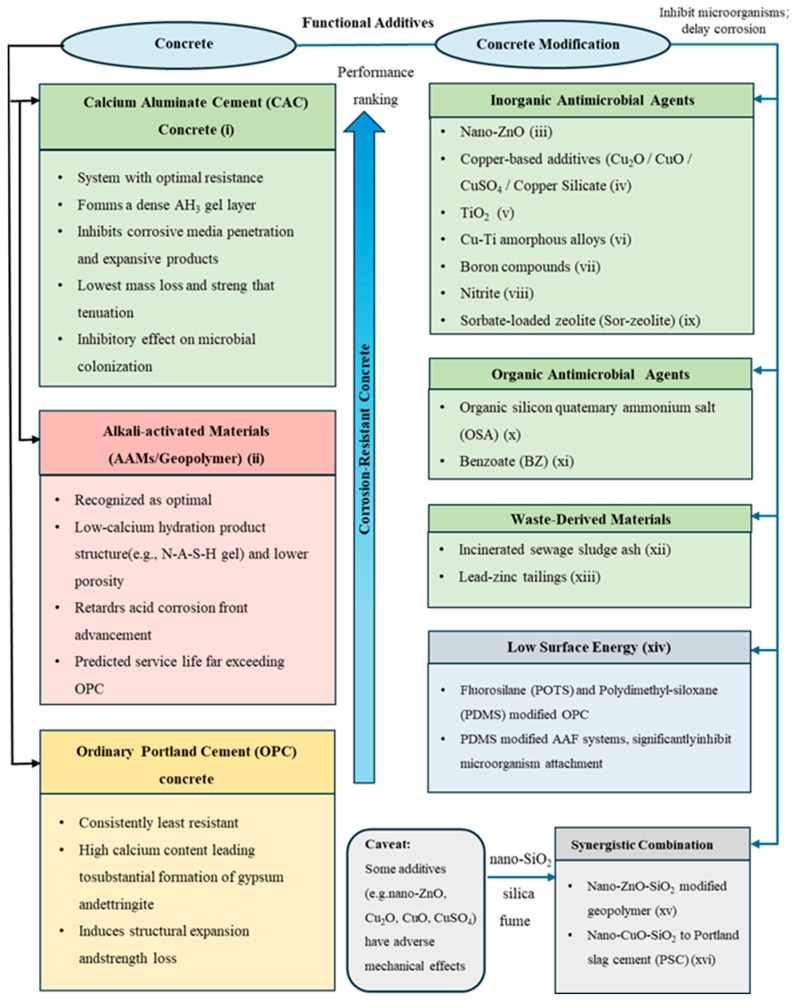
Concrete performance improvement technology for resisting MICC. i: [29,41,103,109]; ii: [27,56,98,106,110]; iii: [83,94]; iv: [77,90,111,112,113]; v: [91,114]; vi: [88,114]; vii: [84]; viii: [115]; ix: [60]; x: [64,81]; xi: [74,76]; xii: [79,116]; xiii: [113]; xiv: [117]; xv: [83]; xvi: [90].

To enhance the resistance of concrete to MICC, strategies focus on two main aspects of the matrix material: first, selecting cementitious materials with inherent tolerance to MICC, and second, modifying the concrete material to impart antimicrobial properties or reduce microbial adhesion (Figure 5).

For corrosion-resistant concrete material, calcium aluminate cement (CAC) concrete and alkali-activated materials are recognized as systems with optimal resistance to microbial corrosion. CAC concrete, due to the formation of a dense AH_3_ gel layer, effectively inhibits the penetration of corrosive media and the formation of expansive products like gypsum. It exhibits the lowest mass loss and strength attenuation in both long-term field exposures and accelerated tests and demonstrates some inhibitory effect on microbial colonization [29,41,103,109]. Alkali-activated materials (AAMs/Geopolymer), owing to their low-calcium hydration product structure (e.g., N-A-S-H gel) and lower porosity, significantly retard the advancement of the acid corrosion front. Their predicted service life in simulated and actual sewage environments far exceeds that of ordinary Portland cement [27,56,98,106,110]. Though CAC and AAMs consistently outperform ordinary Portland cement in laboratory and field studies of MICC resistance, the evidence base has important limitations. Most studies are short-term (<1 year) relative to the intended service life, but the number of long-term field exposures is extremely limited; the range of compositions tested is narrow; and the mechanisms of superior performance are not fully elucidated. For example, while the formation of an AH_3_ gel layer is proposed as the protective mechanism for CAC [29,41,103,109], direct evidence linking AH_3_ formation to reduced microbial colonization or acid penetration is limited. Moreover, the actual resistance of these materials depends critically on multiple interrelated factors, including binder chemistry (e.g., Ca/Si and Al/Si ratios), porosity (influenced by water-to-binder ratio and curing regime), curing conditions (temperature, humidity, and duration), and the specific exposure environment (e.g., pH, temperature, nutrient availability, and microbial community composition). These variables introduce considerable uncertainty when extrapolating laboratory results to field performance, underscoring the need for systematic parametric studies and long-term validation under realistic service conditions.

As for the concrete modification, one approach involves incorporating functional additives, such as antimicrobial agents and mineral admixtures, to enhance microbial corrosion resistance. These improvement strategies, using antibacterial measures, combined with nano-SiO_2_ synergistic effects or by reducing microbial adhesion, both protect concrete from MICC. Based on their primary antimicrobial mechanisms, these additives can be categorized as follows: (i) ion-release agents, including nano-ZnO (which releases Zn^2+^ ions that interfere with bacterial enzymatic functions) [83,94] and copper-based additives such as Cu_2_O, CuO, Cu_2_SO_4_, and copper silicate, which exert biocidal effects through the release of Cu^2+^ ions [77,90,111,112,113]; heavy metal-based bactericidal additives derived from waste materials, such as incinerated sewage sludge ash and lead-zinc tailings, which contain trace heavy metals (e.g., Zn, Cu, Pb) that also inhibit microbial activity through ion release [79,116]; (ii) ROS-generating photocatalysts, such as TiO_2_, which produce reactive oxygen species upon photoexcitation to oxidatively damage microbial cells [91,114], and Cu-Ti amorphous alloys, whose sterilization performance is attributed to both ROS generation and ion release [88,114]; (iii) contact-killing and adhesion-reducing agents, including organic silicon quaternary ammonium salt (OSA), whose silicate component adheres to surfaces to prevent biofilm formation while the ammonium component disrupts bacterial cell walls [64,81], and sorbate-loaded zeolite (Sor-zeolite), which provides sustained antimicrobial release [60]; boron compounds antimicrobial through inhibition of key enzymes, interference with DNA repair mechanisms, and disruption of cell membrane integrity [84]; (iv) pH-modifying agents such as nitrite, which generates free nitrous acid (FNA) under acidic surface conditions to exert biocidal effects [115]; and benzoate (BZ), whose active form—benzoic acid—acts under acidic conditions inhibit microbial [74,76]; and (v) impart low surface energy, fluor silane (POTS) and polydimethylsiloxane (PDMS) modified OPC, and PDMS modified AAF systems all formed superhydrophobic concrete, which significantly inhibits the attachment of microorganisms [116], similar to the hydrophobic coating effect. It should be noted that while the above classification is based on their primary mechanisms, certain additives exhibit multimodal antimicrobial actions. For instance, nano-ZnO not only releases Zn^2+^ ions but also generates ROS under UV irradiation, combining ion-release and photocatalytic effects [83,94]. In addition, multiple modifying agents may be used in combination, for example, nano-ZnO-SiO_2_ decorated geopolymer [83] and Nano CuO-SiO_2_ to Portland slag cement (PSC) [90], in which nano-ZnO and nano CuO inhibit microbial growth and synergistic enhance mechanical properties with nano-SiO_2_.

Concrete modification is a promising protection strategy for MICC; however, the trade-off between antimicrobial efficacy and structural performance is rarely quantified systematically. A critical research need may be the development of design optimization frameworks that balance these competing objectives for specific applications. Furthermore, the long-term fate of antimicrobial additives in the environment—particularly the potential for leaching of toxic metals or nanoparticles—has received insufficient attention. Eco-toxicological assessments should become a standard component of antimicrobial concrete research.

### 4.2. Surface Treatment Technologies

Surface treatment technologies for MICC protection aim to block microbial attachment and the corrosion of the concrete matrix by metabolic products (such as sulfuric acid) through physical isolation, chemical disinfection, or regulation of surface properties. They represent one of the most widely applied techniques in repair and protection. Based on their mechanism of action and material type, these technologies can be categorized into coating protection technologies, electroplating or electrodeposition of metals and metal oxides, etc. (Table 5).

Common coatings primarily include epoxy resins, polyurethanes, and epoxy coal tar pitch coatings. Their main function is to act as a physical barrier, delaying the penetration of corrosive media. However, long-term exposure can lead to failure through multiple mechanisms, including cracking (from mechanical or thermal stress), delamination at the coating–substrate interface, biofilm undergrowth (where microbes penetrate beneath the coating), abrasion, and chemical aging (hydrolysis or UV degradation). Their protective efficacy is influenced by factors such as thickness, fillers, and environmental conditions [118,119].

In recent years, coating protection technologies have been evolving towards multifunctionality, intelligence, and eco-friendliness. A significant development direction is the nano-modification of coatings by incorporating nanoparticles to impart photocatalytic, antibacterial, or densifying properties. For instance, incorporating silver-loaded modified zeolite into polyurethane coatings leverages the sustained release of silver ions (Ag^+^) to inhibit the attachment and metabolism of SOB like *A. thiooxidans* [71]. Alternatively, incorporating MIL-101(Cr) nanoparticles into epoxy resin forms a composite coating that significantly enhances both the bond strength to concrete and resistance to sulfuric acid corrosion [120]. A novel nanocomposite coating has been developed by combining a compatible MoS_2_/MXene heterostructure with polyurea-modified polydimethylsiloxane, exhibiting durable anticorrosion and intelligent antifouling properties [121]. Silica nano-coatings have proven effective in protecting concrete from biodeterioration by *Aspergillus* fungi [47]. Nano-TiO_2_ coatings applied to the surfaces of historical buildings achieve effective inhibition of microbial adhesion. In addition, field monitoring over 4 years showed that areas treated with a TiO_2_/SiO_2_-based product sprayed on ancient building surfaces exhibited a 61% reduction in biofouling compared to untreated areas, demonstrating a lasting antifouling effect [122].

Furthermore, superhydrophobic protective coatings represent another promising strategy. By modifying the surface to achieve low surface energy, these coatings impart strong hydrophobicity to the concrete surface, thereby intercepting the moisture source essential for microbial growth and effectively reducing bacterial adhesion and medium penetration [95]. For example, a fluorine-free polydimethylsiloxane-polymeric silica coating rendered the concrete substrate superhydrophobic, with a water contact angle (WCA) exceeding 150°, along with excellent weather resistance and mechanical durability [123]. Superhydrophobic concrete fabricated using a sacrificial template method, when treated with a mixture of polydimethylsiloxane (PDMS) in cyclohexane and silica nanoparticles, exhibits both a high contact angle (>156°) and mechanical durability, retaining self-cleaning and antibacterial adhesion properties even after abrasion with sandpaper and UV aging [124]. Multifunctional coatings based on an organically modified silica matrix, incorporating TiO_2_ and nano-Ag particles, combine superhydrophobicity, bactericidal action, and photocatalytic activity, effectively inhibiting microbial adhesion in both illuminated and non-illuminated conditions [96]. Additionally, superhydrophobic concrete composites (SCC), prepared by combining epoxy resin with octadecylamine (ODA) to create a micro-nano structure, exhibit superhydrophobicity with a water contact angle of 156° and a sliding angle of 8°, achieving antibacterial rates of 68% against *S. aureus* and 87% against *E. coli* [92].

Electroplating or electrodeposition of metals or their oxides with biocidal activity onto concrete surfaces to form a functional protective layer has emerged as a research hotspot in recent years. Studies have shown that electroplating copper or cuprous oxide (Cu_2_O) onto the surface of cement paste or mortar specimens forms a dense metallic coating that significantly enhances surface antibacterial properties, effectively inhibiting the activity of SRB and SOB [80,125,126,127]. Among these, electrodeposition parameters (such as current density, temperature, pH, and deposition time) significantly influence the coating’s compactness, adhesion, and bactericidal efficacy; excessively high current densities may increase the permeability of the substrate [73,80]. Furthermore, utilizing printed circuit board wastewater as the electrolyte for electrodepositing a Cu@Cu_2_O composite coating not only achieves a high recovery rate (85%) of copper ions from the wastewater but also endows the treated mortar with excellent antibacterial effects against *Desulfovibrio*, SOB, and *E. coli* [78]. However, the sustained release of copper ions from such coatings raises environmental and regulatory concerns, as elevated copper concentrations in aquatic environments are toxic to non-target organisms and may be subject to stringent discharge limits under environmental protection regulations.

Superhydrophobic coatings and electrodeposited metal layers represent promising innovations, but significant technical challenges remain before widespread adoption. For superhydrophobic coatings, the long-term stability of the micro/nanoscale surface texture under mechanical abrasion and chemical attack is a major concern. While some studies report mechanical durability [124], the testing conditions are typically mild compared to real sewer or marine environments where abrasion by suspended solids or wave action occurs. For electrodeposited coatings, the requirement for electrical contact to the reinforcement or external power sources limits applicability to new construction or specific repair scenarios. The scalability and cost-effectiveness of these approaches for large-scale infrastructure have not been demonstrated.

Therefore, we recommend that future studies of surface treatment technologies should include standardized accelerated aging protocols (UV exposure, thermal cycling, abrasion resistance) as a minimum requirement; long-term field exposure at representative sites; systematic characterization of coating failure modes and mechanisms; and cost-benefit analyses comparing coating application to alternative protection strategies. In addition, a suite of standardized microbiological endpoints should be incorporated into performance evaluations, including biofilm biomass (via confocal laser scanning microscopy coupled with Live/Dead staining) [12], viable counts of SOB and SRB (via the most probable number method, MPN), and functional gene abundance (via qPCR, targeting *dsrAB*, *soxB*, and *aprA*) [65], to quantitatively link coating performance to microbial activity.

### 4.3. Ecological Regulation Technologies

Ecological regulation technologies represent an active intervention strategy based on microbial ecology, which inhibits the growth and metabolic activity of corrosive microorganisms by modulating the chemical or biological conditions within the environment (Table 6). Among these, elevating the environmental pH is a commonly employed method. For instance, the addition of alkaline substances such as Mg(OH)_2_ or Ca(OH)_2_ can inhibit the activity of SOB and reduce H_2_S emissions [128,129]. Another approach is competitive inhibition, which involves introducing non-corrosive or beneficial microorganisms to compete with corrosive microbes for nutrients and space, or to form a protective biofilm. For example, utilizing an *E. coli* DH5α biofilm can effectively prevent the attachment and acidifying metabolism influence of SOB [130]. However, it must be emphasized that *E. coli* DH5α is a laboratory strain, not a native environmental isolate; its introduction into real infrastructure is neither practical nor acceptable due to biosafety concerns (e.g., potential pathogenicity, horizontal gene transfer) and environmental release restrictions. Therefore, this approach remains strictly confined to experimental studies. Similarly, loading glycocalyx -producing photosynthetic bacteria like *Rhodopseudomonas palustris* into porous vermiculite and incorporating this into coating mortars enables the continuous generation of a polysaccharide coating that seals microcracks, achieving a protective effect [131]. The biomineralization activity of urease-producing microorganisms can protect concrete through CaCO_3_ precipitation, pore blocking, pH modification, and formation of a physical barrier that limits sulfate diffusion and reduces SRB abundance [20], although its long-term durability remains to be validated. Furthermore, spray with nitrite or free nitrous acid (FNA) can reduce H_2_S generation by inhibiting the metabolism of SRB or by serving as alternative electron acceptors [82,132,133]. Bio-electrochemical systems, such as microbial fuel cells utilizing conductive concrete as electrodes, promote the bio-electrochemical oxidation of H_2_S, thereby mitigating corrosion risk [134]. These ecological regulation methods hold significant application potential; however, their long-term effectiveness, ecological safety, and compatibility with existing building infrastructure require further in-depth investigation.

### 4.4. Critical Comparison of Protection Strategies

The three protection strategies discussed above differ fundamentally in their underlying mechanisms, applicability, and long-term reliability. Corrosion-resistant cementitious materials and surface treatments both aim to create a physical or chemical barrier against microbial attack, whereas ecological regulation technologies seek to actively control the corrosive microbial environment. Their relative merits are highly context-dependent.

Material-level approaches, such as CAC and AAMs, offer built-in durability without requiring post-construction intervention, making them suitable for new infrastructure where life-cycle cost is the primary concern. Yet, their superior performance in laboratory settings—e.g., AAMs exhibit approximately 2.5 times the service life of OPC under simulated conditions [27]—has not been consistently replicated in long-term field exposures, and the protective mechanisms (e.g., AH_3_ gel formation in CAC) remain incompletely understood. Furthermore, the high cost of CAC and the variability in AAM performance due to precursor composition, activator chemistry, and curing conditions limit their widespread adoption. In contrast, surface treatments are versatile and can be applied to existing structures, but their protection is entirely dependent on coating integrity, which is compromised by cracking, delamination, and microbial undergrowth [135]. Among coatings, multifunctional nanocomposite systems (e.g., MIL-101(Cr)/epoxy) show exceptional barrier performance [120], yet they face challenges in scalability, cost, and long-term stability under mechanical abrasion. Superhydrophobic coatings, while offering promising anti-adhesion properties, rely on delicate surface textures that degrade under field conditions [124]. Electrodeposited copper-based coatings exhibit strong bactericidal activity [78,127] but raise environmental concerns due to Cu^2+^ leaching. Ecological regulation technologies, such as nitrite/FNA spraying and biomineralization, offer low-cost, in situ intervention, but their effectiveness is often temporary; the corrosion mitigation from a single nitrite spray diminished within 15 months [136], and the ecological safety of introducing exogenous microorganisms into sewer systems remains largely unaddressed.

A critical knowledge gap across all strategies is the lack of standardized, long-term performance evaluation protocols under realistic conditions. Laboratory-accelerated tests often do not correlate with field performance [97,137], and few studies extend beyond one year, limiting predictions of service life. The environmental footprint of antimicrobial additives, particularly metal ion leaching from materials and coatings [3,138], has been insufficiently investigated, raising concerns about downstream ecological impacts. Future research should prioritize establishing standardized testing frameworks that include microbiological endpoints (biofilm biomass, SOB/SRB viability, functional gene abundance) and developing predictive models that account for the synergistic effects of material chemistry, environmental factors, and microbial community dynamics.

## 5. Concluding Remarks and Perspectives

Although substantial progress has been made in elucidating MICC mechanisms and developing protective materials, several fundamental limitations still hinder the translation of research findings into engineering practice. Future interdisciplinary efforts should focus on the following priority directions.

(1) From single-species mechanisms to complex community dynamics. Current research relying on pure cultures fails to capture multi-species synergies, quorum sensing, and successional shifts. Future work should prioritize advanced experimental platforms integrating multi-omics (metagenomics, metatranscriptomics, metabolomics) with in situ characterization, validated through cross-scale correlation models that translate community dynamics into corrosion prediction.

(2) From isolated microbial corrosion to microbe–environment multi-factor coupling. Most studies neglect coupled abiotic factors such as chloride ingress, carbonation, freeze–thaw cycles, and wetting–drying alternation. Future research should combine in situ multi-scale observations with computational simulations, validated through service life prediction models for diverse environments including sewer networks, marine tidal zones, and industrial cooling systems.

(3) From laboratory efficacy to field-relevant validation and intelligent materials. Existing strategies lack systematic validation of long-term durability, performance degradation under complex conditions, and life-cycle cost-effectiveness. Future development should prioritize intelligent responsive materials (self-healing coatings, conductive concrete, multi-level composite systems), validated through long-term field exposure and comprehensive life-cycle assessment. Moreover, systematic eco-toxicological assessments of antimicrobial additives are essential to ensure environmental sustainability.

(4) From unreinforced laboratory specimens to reinforced concrete systems. The synergistic interaction between steel reinforcement corrosion and MICC deterioration remains severely understudied, as most studies ignore the coupled electrochemical–mechanical–biochemical processes in real structures. Future research should prioritize integrated experimental–computational frameworks coupling reinforcement corrosion with MICC deterioration, validated through long-term exposure of reinforced concrete in real sewer and marine environments.

## Figures and Tables

**Table 1:** Comparison of corrosive and protective biofilms in marine concrete.

Feature	Corrosive Biofilms	Protective Biofilms
Dominant microorganisms	SOB (*Thiobacillus* spp., *Acidithiobacillus* spp.), SRB	*Pseudoalteromonas* sp. B18, *Paracoccus marcusii* B23, other biofilm-forming heterotrophs
Biofilm characteristics	Typically thin, patchy, or heterogeneous; may be disrupted by environmental stresses	Dense, mature, and continuous; well-established EPS matrix
Key mechanisms	Biogenic acid production (H_2_SO_4_, organic acids); sulfide oxidation; gypsum and ettringite formation	Diffusion barrier against Cl^−^, Mg^2+^, and other aggressive ions; reduced OH^−^ leaching; biomineralization (CaCO_3_ precipitation) sealing surface pores
Effect on concrete	Accelerated decalcification, increased permeability, surface cracking, and spalling	Reduced ion penetration, maintained alkalinity, decreased corrosion rate, extended service life

**Table 4:** General antimicrobial indicator microorganisms used in protective material testing.

Microbial Category	Genus/Species	References
Gram-negative bacterium	*Escherichia coli*	[78,83,88,89,90,91,92,93,94,95,96]
	*Pseudomonas* spp.	[94]
Gram-positive bacterium	*Staphylococcus aureus*	[83,90,92,93,94]
	*Bacillus subtilis*	[89]
Fungi/Yeast	*Candida albicans*	[84]
	*Saccharomyces cerevisiae*	[95,96]

**Table 5:** Classification and performance comparison of surface treatment technologies for microbial corrosion protection of concrete.

Category	Typical Example	Potential Mechanism	Cost	Scalability	Maintenance	References
Organic coating	Epoxy resin, polyurethane, epoxy coal tar pitch coating	Physical barrier function delays the penetration of corrosive media	Moderate	High	Moderate (periodic reapplication required)	[118,119]
Nano-modified coating	Ag^+^-loaded zeolite-polyurethane coating	Release of Ag^+^	High	Moderate	Low (long-term Ag^+^ release efficacy uncertain)	[71]
	nano-MIL-101(Cr) epoxy composite coatings	Enhance the chemical cross-linking of the epoxy matrix and increase adhesion strength	High	Moderate	Low	[120]
	MoS2/MXene heterostructure polyurea-modified PDMS coating	MoS_2_/MXene provides contact bactericidal action, and photocatalysis generates ROS for sterilization	High	Low	Moderate	[121]
	silicon dioxide nanocoating	Physical barrier and chemical stability	Moderate	High	Low	[47]
	A bare TiO_2_/SiO_2_ treatment	Photocatalysis generates ROS for sterilization	Moderate	High	Moderate	[122]
Superhydrophobic coating	Polydimethylsiloxane and polymeric silica fluorine-free superhydrophobic coating	Low surface energy	Moderate	Moderate	High (texture susceptible to abrasion)	[123]
	Polydimethylsiloxane, cyclohexane, and silica nanoparticles mixture	Formed superhydrophobic concrete	Moderate	Moderate	High	[124]
	Functionalized Ag-TiO_2_NPs to ormosil-based coatings	superhydrophobic, release of Ag^+^, and photoactive properties	High	Low	Moderate	[96]
	Octadecylamine modified epoxy resin coating	Low surface energy	Moderate	High	Moderate	[92]
Metal/metal oxide deposition	electroplated layer of Cu, Cu_2_O, Cu@Cu_2_O	Sustained-release sterilization of Cu^2+^	High	Low	Low (limited to new construction/repair scenarios)	[80,125,126,127]

**Table 6:** Classification and performance comparison of ecological control technologies for MICC protection.

Category	Typical Example	Potential Mechanism	References
pH control	Add NaOH or Mg(OH)_2_	Reduce the H_2_S fraction of dissolved sulfide	[128,129]
Microorganisms control	*E. coli* DH5α biofilm	Compete for attachment space and form a protective barrier	[130]
	Glycocalyx-producing photosynthetic bacteria, like *Rhodopseudomonas* *palustris*	Produce glycocalyx	[131]
	Biomineralization from urease-producing bacteria (UPB)	Biomineralized film decreases the abundance of SRB and form a protective layer	[20]
Chemical addition	Spray with nitrite or free nitrous acid (FNA)	Alternative electron acceptor and multiple-target antimicrobial effects	[82,132,133]
Bio-electrochemistry	Conductive concrete and microbial fuel cell (MFC)	Methanogens actively consumed sulfide, supported by enhanced electron transfer	[134]

## Data Availability

No new data were created or analyzed in this study.

## Abbreviations

The following abbreviations are used in this manuscript:

MICC	Microbiologically induced concrete corrosion
SOB	Sulfur-oxidizing bacteria
SRB	Sulfate-reducing bacteria
NSOB	Neutrophilic sulfur-oxidizing bacteria
ASOB	Acidophilic sulfur-oxidizing bacteria
AFt	Ettringite
C-S-H	Calcium silicate hydrate
CAC	Calcium aluminate cement
PSC	Portland slag cement
AAMs	Alkali-activated materials
OSA	Organic silicon quaternary ammonium salt
WCA	Water contact angle
